# Radiological-Pathological Correlation in Plasmablastic Lymphoma in an Immunocompromised Patient

**DOI:** 10.1155/2018/4746050

**Published:** 2018-10-21

**Authors:** A. Moramarco, M. Marenco, M. La Cava, A. Lambiase

**Affiliations:** Department of Sense Organs, Sapienza University of Rome, Italy

## Abstract

Plasmablastic lymphoma (PBL) is a malignant, rare, and aggressive form of non-Hodgkin's lymphoma with poor response to treatment that most commonly involves the oral mucosa of immunodeficient patients. The orbit might be primarily or secondarily involved; on the other hand other pathological conditions, i.e., fungal infections, may localize in the orbit in both immunocompromised patients and drug user, which might have similar radiological features. We describe the clinical, radiological, and pathological features of an orbital plasmablastic lymphoma (PBL) in an immunocompromised HIV positive drug user patient.

## 1. Introduction

Plasmablastic lymphoma (PBL) is a very aggressive variant of diffuse large B-cell lymphoma initially described in the oral cavity of immunocompromised patients, recently considered by WHO classification as a separated entity under mature B-cell neoplasms with a distinct clinical and immunohistochemical profile [1]. Plasmablastic lymphoma is frequently resistant to therapy and often rapidly fatal [1, 2]. Ocular involvement is extremely rare with very few cases reported in the literature [3–7]. We present a case of orbital plasmablastic lymphoma whose MRI characteristics might be observed in different entities that have to be considered in the differential diagnosis for orbital mass lesions.

## 2. Case Presentation

**Figure 1:**
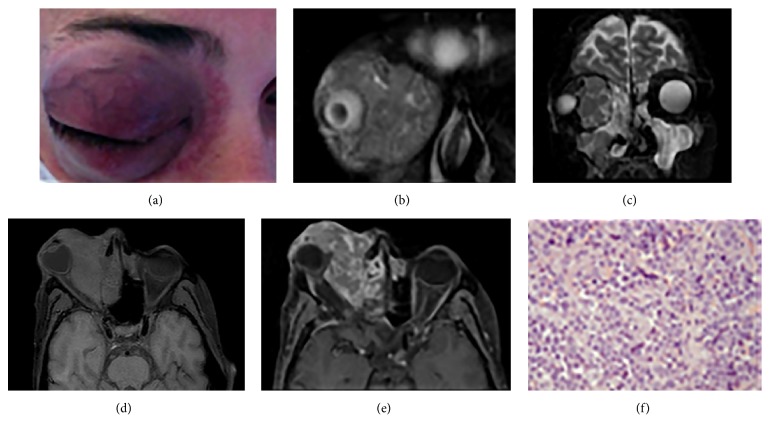
At clinical examination the patient shows proptosis, downward displacement of eye, periorbital swelling, and redness (a). MRI fat-suppressed coronal T2-weighted (b, c) and axial T1-weighted images (d) show an inhomogeneous hypointense mass located in the lateral aspect of the right orbit that extends to the nose and ethmoid sinus with intense and heterogeneous contrast enhancement (e). H&E stain of the tumor shows a monomorphic proliferation of plasmablastic cells (f).

A 35-year-old man was admitted at the oculistic emergency room for a right exophthalmos accompanied with severe pain, periorbital swelling, redness, and mucopurulent discharge. The patient, HIV and HCV positive for 11 years, had a history of drug and alcohol addiction, including habitual marijuana smoking. Clinical examination revealed severe nonreducible exophthalmos with bulb dislocated medially and inferiorly, associated with eyelid chemosis and decreased vision in the right eye (1/10). The motility of the globe in all gazes was restricted by mechanical proptosis. Fundus examination revealed optic disc swelling associated with venous congestion. A brain MRI was performed in order to evaluate the involvement of the orbit and the brain. The examination showed an intraorbital mass that was inhomogeneously hypointense on T2-weighted images and hypointense on T1-weighted images and showed intense and inhomogeneous contrast enhancement. The mass was located in the lateral orbit cavity extending to the ethmoid and maxillary sinuses, with bony destruction, encasing and displacing the medial rectus and superior oblique muscles, optic nerve, ocular globe, and vascular structures (Figure 1).

On the basis of these findings and considering patient's history of HIV related diseases and drug addiction, few diagnosis have been hypothesized that included neoplastic diseases, e.g., lymphoma, and infections, in particular mycotic origin, such as aspergillosis, considering the known association with marijuana smoking. Prior to beginning any treatment, the patient was subjected to a biopsy of the orbital mass. At histology H&E stain of the tumor showed a monomorphic proliferation of plasmablastic cells that was suggestive of plasmablastic lymphoma. The patient died within a week following diagnosis for a heart attack, even before further treatment could be initiated.

## 3. Discussion

Plasmablastic Lymphoma (PBL) is an aggressive and malignant form of non-Hodgkin's lymphoma. Recently the World Health Organization (WHO) has recognized plasmablastic lymphoma as a different entity under mature B-cell neoplasm [1, 2]. PBL is more commonly associated with preceding immunodeficiency and accounts for 2.6% of all HIV related non-Hodgkin's lymphoma [2]. PBL mean age at presentation in HIV patient is 44.8 years and it is more common in men than women. PBL is frequently resistant to therapy and often rapidly fatal [2]. The most common localizations are extra-nodal sites, usually the oral cavity and the jaw; the ocular involvement is extremely rare. Few cases had been described so far in which there is a primary involvement of the orbit [3–7]. MRI findings of our case correspond to those found on the literature; consisting on an intermediate signal on T1-weighted images and iso-hyperintense signal on T2-weighted images with variable postcontrast enhancement [3–7]. Nevertheless these nonspecific MRI findings are similar to the ones that can be encountered in other orbital pathology such as infective diseases. On MRI, fungal sinusitis shows inhomogeneous hypointense signal on T2-weighted images due to the presence of increased concentrations of iron and calcium in fungal concretion [8]. Furthermore in our case the patient's history of drug addiction, including marijuana smoking and HIV related diseases, as well the involvement of the paranasal sinuses, prompted consideration of mycotic and other infectious diseases together with neoplastic diseases, including lymphoma and sarcoma. Fungal infections in paranasal sinus are common in patients with HIV. Habitual marijuana smoking may increase the risk for development of sino-orbital aspergillosis in these patients due to the contamination of the smoking materials [9]. It occurs as a direct extension of infection of the paranasal sinuses. Therefore in this case mycosis was highly suspected. MRI features of our case mimic an inflammatory process, thus making PBL a diagnostic challenge especially for immunocompromised patients in which both entities have to be considered. Differentiation between fungal sinusitis and plasmablastic lymphoma is therefore important for the outcome and the therapeutic strategy. Even though definitive diagnosis is based on histopathological investigation, imaging should be performed in all cases, and MRI scanning is considered being superior to other imaging modalities. When inflammatory or infectious disease is suspected, proper laboratory tests should be conducted as well.
